# PERIODIC FASTING MIMICKING DIET, LONGEVITY, AND DISEASE

**DOI:** 10.1093/geroni/igac059.362

**Published:** 2022-12-20

**Authors:** Valter Longo

**Affiliations:** Leonard Davis School of Gerontology, Los Angeles, California, United States

## Abstract

Chronic dietary interventions have been known for decades to help prevent disease and extend longevity, yet most are difficult to adopt especially long-term. Brief periods of a diet that mimics fasting by regulating key starvation response genes including IGF-1, TOR-S6K and PKA lasting between 4 and 7 days and followed by long periods on a normal diet, are emerging as potentially effective pro-longevity interventions. These periodic fasting mimicking diets (FMD) provide low calories, sugars, and proteins and high levels of unsaturated fats. In mice, 4 day bi-monthly cycles of the FMD started at middle age extend longevity, reduce tumors by nearly 50%, reduce inflammatory diseases and increase cognitive performance at old ages. In humans, 3 monthly cycles of a 5 day FMD reduce markers or risk factors for aging, diabetes, cancer and cardiovascular disease including cholesterol, blood pressure, CRP, IGF-1, and fasting glucose, particularly in subjects with elevated levels of these markers at baseline. Here I will present our most recent mouse and clinical studies indicating that FMDs can help reverse insulin resistance, reduce risk factors/markers of aging and age-related diseases, and lower biological age.

